# KLF15 Regulates Oxidative Stress Response in Cardiomyocytes through NAD^+^

**DOI:** 10.3390/metabo11090620

**Published:** 2021-09-13

**Authors:** Le Li, Weiyi Xu, Lilei Zhang

**Affiliations:** Department of Molecular and Human Genetics, Baylor College of Medicine, Houston, TX 77004, USA; lile11@126.com (L.L.); weiyix@bcm.edu (W.X.)

**Keywords:** ROS, oxidative stress, cardiomyocytes, KLF15, NAD^+^

## Abstract

KLF15 has recently emerged as a central regulator of metabolism. Although its connection to oxidative stress has been suspected, there has not been any study to date that directly demonstrates the molecular link. In this study, we sought to determine the role of KLF15 in cardiac oxidative stress. We found that KLF15 deficiency in the heart is associated with increased oxidative stress. Acute deficiency of KLF15 in neonatal rat ventricular myocytes (NRVMs) leads to the defective clearance of reactive oxygen species (ROS) and an exaggerated cell death following a variety of oxidative stresses. Mechanistically, we found that KLF15 deficiency leads to reduced amounts of the rate-limiting NAD^+^ salvage enzyme NAMPT and to NAD^+^ deficiency. The resultant SIRT3-dependent hyperacetylation and the inactivation of mitochondrial antioxidants can be rescued by MnSOD mimetics or NAD^+^ precursors. Collectively, these findings suggest that KLF15 regulates cardiac ROS clearance through the regulation of NAD^+^ levels. Our findings establish KLF15 as a central coordinator of cardiac metabolism and ROS clearance.

## 1. Introduction

The mammalian heart has a high energy need for the support of its contractile function. About 70% of the energy is converted from fatty acid oxidation at rest [1]. Reactive oxygen species (ROS) is an intrinsic by-product of oxidative phosphorylation, and it is inevitably produced with oxidative metabolism from the mitochondrial electron transport chain. Thus, it may be beneficial for the organism to coordinate catabolism and ROS clearance. However, such a molecular coordinator has not been characterized.

The Kruppel-like factor (KLF15) is a zinc finger transcription factor with a central role in nutrient regulation in key metabolic organs, including the liver, skeletal muscle, fat, and the heart [2,3,4,5,6,7]. In the myocardium, KLF15 directly controls the lipid flux at multiple levels, which is the main energy source at rest [5,8]. KLF15 also transcriptionally regulates additional catabolic pathways in the heart, e.g., multiple amino acid degradation [9]. KLF15-deficient mice exhibit heightened cardiac hypertrophy and heart failure after both pressure overload and neurohormonal stress [9,10]. Furthermore, we recently showed that cardiac KLF15 deficiency is associated with increased ischemia and reperfusion injury during the active phase, suggesting that KLF15 has a protective role in oxidative injury [11]. However, the exact molecular mechanisms remain unexplored.

Nicotinamide adenine dinucleotide or NAD^+^ plays a vital role in both cellular metabolism and redox homeostasis [12,13]. The reduction of NAD^+^ to NADH occurs during fuel catabolism. The β-oxidation of fatty acids and the tricarboxylic acid (TCA) cycle reduce NAD^+^ to NADH. NADH is then fed into the electron transport chain to generate ATP in the mitochondria while being oxidized back to NAD^+^. In the heart, NAD^+^ is primarily generated by the salvage pathway, which converts NAD^+^ from nicotinamide (NAM) via NAM phosphoribosyltransferase (NAMPT)—the rate-limiting enzyme [14,15,16]. Dysregulation of NAD^+^ homeostasis is associated with a number of diseases, including cardiovascular diseases (CVD) [13,16,17,18,19]. Despite the key role of NAD^+^ in cardiovascular health, how it coordinates catabolism and ROS clearance has not been elucidated.

Here, we provide evidence that KLF15 regulates ROS clearance by transcriptionally activating the NAD^+^ producing enzyme NAMPT and controls the level of cardiomyocytic NAD^+^, which subsequently affects global mitochondrial acetylation and the activity of ROS clearance antioxidants, e.g., MnSOD. Thus, we identified KLF15 as the transcriptional hub that coordinates the regulation of both cardiac catabolism and ROS clearance.

## 2. Results

### 2.1. KLF15 Regulates Myocardial ROS

**Figure 1:**
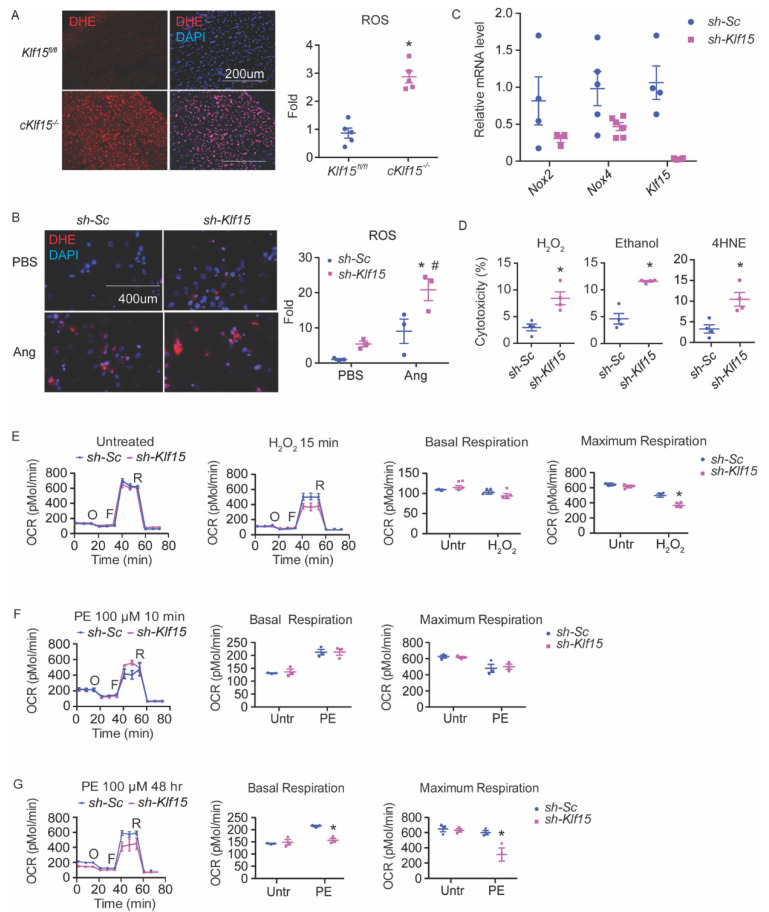
KLF15 deficiency leads to increased susceptibility to ROS in the cardiomyocytes. (**A**) ROS in the hearts of cKlf15 KO and litter mate controls (8–10 weeks old, male) was measured by DHE staining (red) and DAPI (blue), and quantified using NIH Image J (*n* = 5, *: *p* < 0.0001 vs. litter mate controls). (**B**) NRVMs with shRNA knockdown of scrambled RNA (*sh-Sc*) or *Klf15* (*sh-Klf15*). ROS was induced by treatment with Angiotensin II for 24 h and was measured by DHE staining (red) and DAPI (blue), and quantified using NIH Image J (*n* = 3, *: *p* < 0.01 vs. *sh-Sc* without treatment; #: *p* < 0.01 vs. *sh-Klf15* without treatment). (**C**) qRT-PCR of *Nox2*, *Nox4,* and *Klf15* in NRVM with *sh-Sc* or *sh-Klf15* (*n* = 3–6). (**D**) NRVMs with shRNA knockdown of scrambled RNA (*sh-Sc*) or *Klf15* (*sh-K15*) were subjected to different stimuli. Percentage of cytotoxicity derived from LDH assay after treatment with each stimulus is shown (ethanol: 100 mM 6 h; H_2_O_2_: 100 μM 2 h; 4HNE: 10 μM 8 h) (*n* = 4, *: *p* < 0.05 vs. *sh-Sc*). (**E**–**G**) Mitochondrial ETC function in NRVM assessed by Seahorse Mito Stress test using (**E**) H_2_O_2_ for 15 min, (**F**) phenylephrine (PE) for 10 min, (**G**) PE for 48 h, respectively (*n* = 3, *: *p* < 0.05 vs. *sh-Sc*, two-tailed Student’s *t*-test). The Holm–Sidak method was used to correct for multiple *t*-tests with alpha set as 0.05. Data are presented as mean ± SEM.

Previously, we reported that KLF15 directly regulates a number of catabolic enzymes [9]. ROS are intrinsic byproducts of metabolism. We speculated that KLF15 may also play a role in the disposal of ROS from catabolism. To test this hypothesis, we sought to investigate if KLF15 deficiency in the cardiomyocytes is associated with increased ROS. We assessed the ROS levels by dihydroethidium (DHE) staining in flash-frozen heart tissues from a cardiomyocytic-specific KLF15 null mice (cKlf15 KO, cKlf15flox/flox:α-MHC-cre). The KLF15-deficient myocardium showed an increased ROS, suggesting that KLF15 regulates myocardium ROS (Figure 1A).

### 2.2. KLF15 Depletion Increases the Susceptibility to Oxidative Stress in the Cardiomyocytes, Likely Due to Reduced ROS Clearance

To dissect the underlying mechanism for the increased myocardial ROS in the cKlf15 KO mice, we studied in vitro the cellular response to oxidative stress of the NRVMs sufficient and deficient in KLF15. Similar to the observation in cKlf15 KO mice heart, KLF15-deficient NRVMs showed a pronounced increase of ROS after treatment with angiotensin II (Figure 1B). Angiotensin II induces ROS through NADPH oxidase (NOX) [20]. The expression of both Nox2 and Nox4, the main NOX isoforms, in the heart showed a downward trend in the KLF15-deficient cells but not up, suggesting that increased ROS production is unlikely to be due to the upregulation of NOX (Figure 1C). In addition to NOX, an abnormal electron transport chain (ETC) may also increase ROS production. Using pyruvate as a substrate, both basal and maximal oxygen consumption in KLF15-deficient NRVMs are indistinguishable from those of the control cells at baseline (Figure 1E). These results suggest that increased ROS production is unlikely to be the major cause of ROS imbalance in KLF15-deficient cardiomyocytes.

The acute silencing of *Klf15* in NRVMs led to an enhanced sensitivity to several different oxidants measured by lactate dehydrogenase (LDH) assay, including hydrogen peroxide (H_2_O_2_), 4-hydoxyl-2-nonenal (4HNE, a major lipid peroxidation product), and alcohol (which leads to increased intracellular aldehyde) (Figure 1D). A brief treatment of H_2_O_2_ for 15 min (prior to any appreciable cell death) led to decreased respiratory capacity in KLF15-deficient cells, suggesting an exaggerated ETC dysfunction from ROS injury. Furthermore, while KLF15-deficient NRVMs responded to a short treatment (15 min) of phenylephrine (PE), comparable to that of the control cells, prolonged treatment of PE (48 h), which is known to augment oxidative stress [21], led to reduced respiratory capacity (both basal and maximum respiration) in KLF15-deficient cells, similar to post-treatment with H_2_O_2_ (Figure 1E–G). These results suggest that the observed ROS imbalance in KLF15-deficient cardiomyocytes is most likely due to poor clearance.

### 2.3. Tempol Reverses the Cellular Defect of KLF15 Deficiency during Oxidative Stress

**Figure 2:**
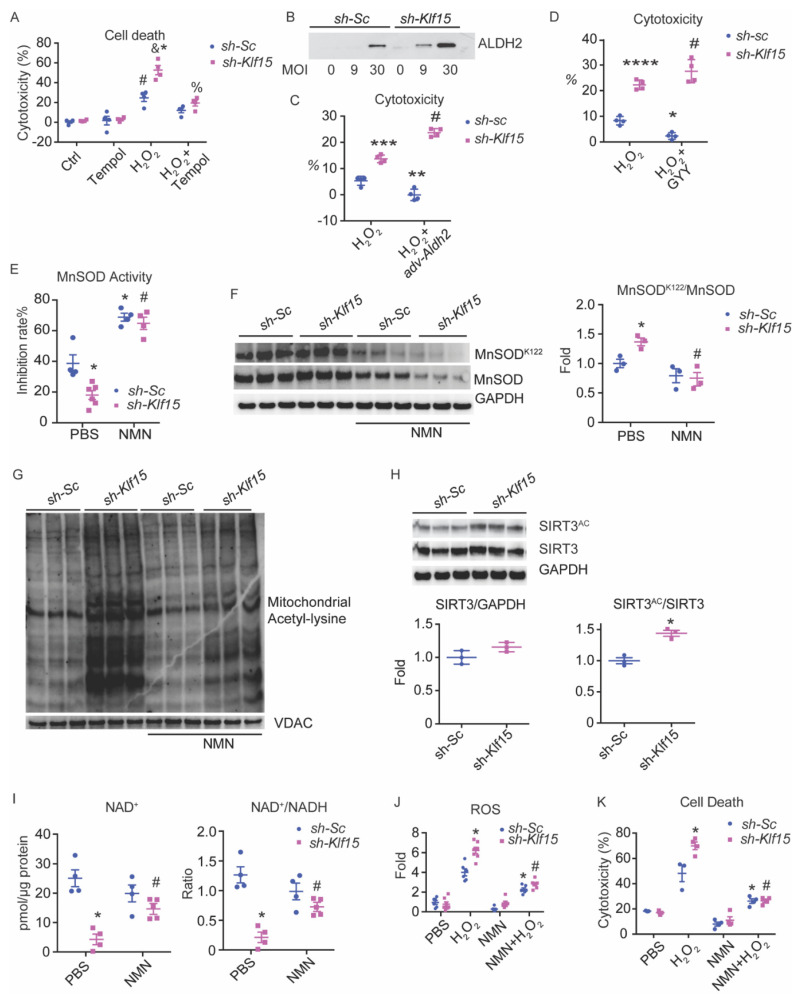
KLF15 deficiency leads to increased susceptibility to ROS due to NAD^+^ deficiency. NRVMs were treated with shRNA knockdown of scrambled RNA (*sh-Sc*) or *Klf15* (*sh-Klf15*). (**A**) NRVMs were subjected to 3 mM Tempol for 10 min prior to 100 μM H_2_O_2_ for 2 h. Percentage of cytotoxicity derived from LDH assay after treatment is shown. (*n* = 4, *: *p* < 0.0001 vs. *sh-Sc* without treatment; #: *p* < 0.04 vs. *sh-Sc* without treatment; &: *p* < 0.001 vs. *sh-Sc* treated with H_2_O_2_; %: *p* < 0.0002 vs. *sh-Klf15* treated with H_2_O_2_). (**B**) Ectopic ALDH2 expression by adenoviral vector in NRVMs detected by immunoblotting. (**C**) NRVMs were infected with control adenoviral vector (*adv-Gfp*) or *adv-Aldh2* for 48 h. Cell death was then induced by 100 μM H_2_O_2_ for 90 min before measurement by LDH assay. (*n* = 3–4, **: *p* < 0.01, ***: *p* = 0.0001 vs. *sh-Sc* treated with H_2_O_2_ and infected with *adv-Gfp*, #: *p* < 0.0001 vs. *sh-Sc* treated with H_2_O_2_ and infected with *adv-Aldh2*). (**D**) NRVMs were treated with H_2_S stable releaser GYY4137 or vehicle and then stimulated with 100μM H_2_O_2_ for 90 min. Percentage of cytotoxicity derived from LDH assay. (*n* = 3, *: *p* < 0.05, ****: *p* < 0.0001 vs. *sh-Sc* treated with H_2_O_2_, #: *p* < 0.0001 vs. *sh-Sc* treated with H_2_O_2_ and GYY4137). (**E**–**K**) NRVMs were treated with 2 mM NMN or PBS for 24 h. (**E**) MnSOD activity in NRVMs (*n* = 4–6, *: *p* < 0.01 vs. *sh-Sc* treated with PBS, #: *p* < 0.001 vs. *sh-Klf15* treated with PBS). (**F**) MnSOD and MnSOD^K122^ acetylation levels were measured by immunoblot and the ratio was calculated based on densitometry (*n* = 3, *: *p* < 0.01 vs. *sh-Sc* treated with PBS, #: *p* < 0.001 vs. *sh-Klf15* treated with PBS). (**G**) Mitochondrial total protein acetylation was measured using anti-acetyl-Lysine immunoblot blot. VDAC was used as a loading control for mitochondrial protein. (**H**) SIRT3 and GAPDH protein levels were determined by immunoblot. Acetylation of SIRT3 was determined by immunoblot for anti-acetyl-Lysine after immunoprecipitation with SIRT3 antibody. Quantified ratio was derived from densitometry. (*n* = 3, *: *p* = 0.003, two-tailed Student’s *t*-test). (**I**) NAD^+^ level and NAD^+^/NADH ratio. (*n* = 4, *: *p* < 0.001 vs. *sh-Sc* treated with PBS; #: *p* < 0.05 vs. *sh-Klf15* treated with PBS). (**J**,**K**) 100μM H_2_O_2_ for 90 min was used to induce oxidative stress. (**J**) ROS was measured by DHE staining and quantified using NIH Image J (*n* = 6, *: *p* < 0.001 vs. *sh-Sc* treated with H_2_O_2_; #: *p* < 0.0001 vs. *sh-Klf15* treated with H_2_O_2_). (**K**) Percentage of cytotoxicity derived from LDH assay (*n* = 3–4, *: *p* < 0.0001 vs. *sh-Sc* treated with PBS; #: *p* < 0.0001 vs. *sh-Klf15* treated with H_2_O_2_). Two-way ANOVA with Bonferroni correction was used to determine statistic difference. Data are presented as mean ± SEM.

To further verify that the exaggerated cell death in KLF15-deficient cells are indeed due to the inability to handle oxidative stress and are not due to other unhealthy states of the cells, we pretreated the NRVMs with Tempol for 10 min and performed the H_2_O_2_-induced cell death assay. Tempol is a superoxide dismutase (SOD) mimetic, which converts O_2_^−^ to H_2_O_2_. As predicted, there was a complete rescue of the previously observed exaggerated cell death by Tempol, confirming our hypothesis that KLF15-deficient cardiomyocytes are more susceptible to oxidative injury due to defective ROS handling (Figure 2A). To further evaluate whether the observed Tempol rescue was specific or was a more general antioxidant effect, we attempted similar rescues using several known antioxidant KLF15 targets, including Aldehyde dehydrogenase 2 (*Aldh2*) and Cystathionine gamma lyase (*Cth*). Interestingly, both only protected the control cells as expected and not the Klf15 knock-down cells (Figure 2B–D). The lack of benefit of these targets suggests that they did not provide the specific rescue for the molecular defect of KLF15-deficient cardiomyocytes during oxidative injury.

### 2.4. KLF15 Deficiency Resulted in NAD^+^ Deficiency and Subsequent Hyperacetylation of Mitochondrial Proteins and Reduced Activity of MnSOD

Since Tempol provided the specific rescue for oxidative stress in KLF15-deficient cardiomyocytes, we suspected that SOD deficiency might be an important mediator of the observed increase in ROS and the increased susceptibility to oxidative stress in the absence of KLF15. Further strengthening this hypothesis, we found reduced MnSOD activity in the KLF15-deficient NRVMs (Figure 2E). As KLF15 is a transcription factor with numerous metabolic targets, we first examined the MnSOD expression level. To our surprise, MnSOD protein expression was unchanged. Instead, we found hyperacetylation at MnSOD^K122^ in KLF15-deficient NRVMs, which is associated with reduced MnSOD activity (Figure 2F). Furthermore, we observed a global hyperacetylation of mitochondrial proteins in the acutely KLF15-deficient NRVMs (Figure 2G). SIRT3 is the main de-acetylase in the mitochondrial matrix [22]. Interestingly, SIRT3 level was also unchanged, but with hyperacetylation (Figure 2H). We next assessed the level of its co-enzyme NAD^+^. Indeed, in KLF15-deficient NRVMs, there was a dramatically reduced NAD^+^ and the NAD^+^/NADH ratio (Figure 2I). Together, these results suggest that the absence of KLF15 is associated with NAD^+^ deficiency, which then leads to SIRT3 dysfunction and the hyperacetylation of mitochondria proteins, including MnSOD, and subsequently leads to reduced activity of MnSOD and likely other mitochondrial proteins.

### 2.5. KLF15 Deficiency Leads to Reduced Nampt

**Figure 3:**
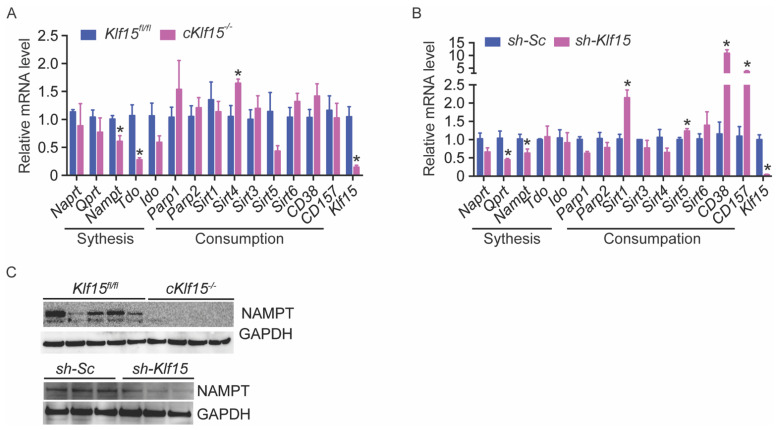
KLF15 deficiency leads to reduced expression of *Nampt*. (**A**,**B**) Quantitative RT-PCR results of NAD^+^ synthetic and consuming enzymes in both the cKlf15 KO mice hearts (**A**) and the acute KLF15-deficient NRVM model (**B**) (*n* = 3–4, *: *p* < 0.05 vs. littermate controls or *sh-Sc*, two-tailed Student’s *t*-test). Holm–Sidak method was used to correct for multiple t-tests, with alpha set as 0.05. Data are presented as mean ± SEM. (**C**) NAMPT protein expression in cKlf15 KO mice and littermate control hearts (top, *n* = 4–5) and in NRVM with shRNA knockdown of scrambled RNA (*sh-Sc*) or *Klf15* (*sh-Klf15*) (bottom, *n* = 3). GAPDH was used as loading control.

To investigate the possible molecular mechanisms that lead to NAD^+^ deficiency in the absence of KLF15, we examined NAD^+^ biosynthesis enzymes, including salvage pathways, as well as NAD^+^ consumption enzymes using qRT-PCR in both the acute KLF15-deficient NRVM model and the cKlf15 KO mice hearts (Figure 3A,B). The only change that occurred in both systems and was also consistent with the reduced level of NAD^+^ in the absence of KLF15 was the reduction of *Nampt*. Reduced NAMPT protein was also observed in the cKlf15 KO heart and in the acute *Klf15* knockdown NRVMs (Figure 3C). NAMPT was reported to be the determinant factor of cardiac NAD^+^, and our result suggests that KLF15 may regulate cardiomyocytic NAD^+^ through *Nampt* at the transcript level (reduced mRNA expression level).

### 2.6. NMN Rescued the KLF15 Deficiency-Associated Susceptibility to Oxidative Stress

Given the pronounced reduction in NAD^+^, we attempted to provide a rescue by supplementing the cell and mitochondria permeable NAD^+^ precursor, nicotinamide mononucleotide (NMN). We added 2 mM NMN to the culture medium for 24 h, which corrected the reduction in NAD^+^ and the NAD^+^/NADH ratio (Figure 2I). The global hyperacetylation of mitochondrial protein was also ameliorated in the KLF15-deficient NRVMs, including the hyperacetylation of MnSOD^K122^ (Figure 2F,G). More importantly, pretreatment with NMN for 24 h prior to injury reduced the H_2_O_2_-induced ROS as well as the cell death in KLF15-deficient NRVMs to similar levels as the control cells, suggesting that the NAD^+^ deficiency was indeed the primary defect leading to exaggerated injury from oxidative stress (Figure 2J,K).

### 2.7. The Cardioprotective Effect of NMN in Oxidative Stress Is SIRT3-Dependent

**Figure 4:**
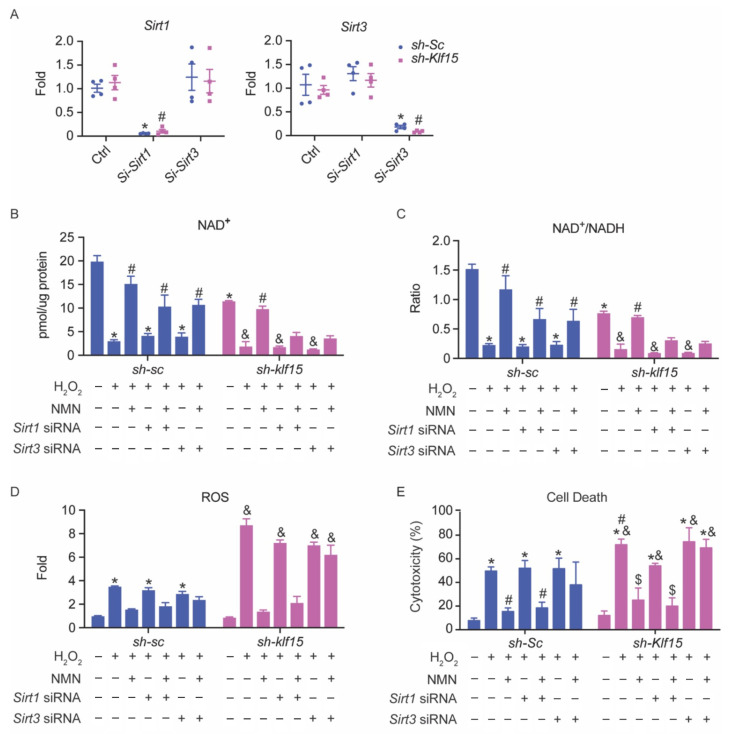
The cardioprotective effect of NMN in oxidative stress is SIRT3-dependent. NRVMs with shRNA knockdown of scrambled RNA (*sh-Sc*) or *Klf15* (*sh-Klf15*) were transfected with 25 nM *Sirt1*, *Sirt3* siRNA, or negative control siRNA for 48 h. (**A**) qPCR analysis of *Sirt1* and *Sirt3* levels in NRVMs (*n* = 4, *: *p* < 0.001 vs. NRVM transfected with negative control siRNA and *sh-Sc*; #: *p* < 0.001 vs. NRVM transfected with negative control siRNA and *sh-Klf15*). (**B**) NRVMs were pretreated with 2 mM NMN or PBS for 24 h prior to exposure to 100 μM H_2_O_2_ for 90 min. (**B**) NAD^+^ level. (**C**) NAD^+^/NADH ratio. (**D**) ROS was measured by DHE fluorescence intensity in a microplate reader, with excitation at 485 nm and emission at 590 nm. The fluorescence intensity was normalized to total protein in each sample. (**E**) Percentage of cytotoxicity was derived from LDH assay. (*n* = 3–4, *: *p* < 0.01 vs. *sh-Sc* without treatment; #: *p* < 0.05 vs. *sh-Sc* treated with H_2_O_2_; &: *p* < 0.01 vs. *sh-Klf15* without treatment; $: *p* < 0.05 vs. *sh-Klf15* treated with H_2_O_2_). Two-way ANOVA with Bonferroni correction was used to determine statistic difference. Data are presented as mean ± SEM.

Previously, NAD^+^ was reported to play a protective role during cardiac I/R in a SIRT1-dependent fashion [23]. Yet, our results suggest that it also reduces mitochondrial acetylation and promotes mitochondrial protein function during oxidative stress associated with SIRT3 function. To investigate if the NMN rescue of oxidative stress in KLF15 deficiency is SIRT1 or SIRT3-dependent, we achieved an acute knockdown in NRVMs using siRNA, specifically targeting SIRT1 or SIRT3 (Figure 4A). To our surprise, in the absence of SIRT1, the NMN rescue by cell death or ROS levels were not affected (there was a mild trend towards reduction in the control cells, but the difference was not significant). In contrast, in the absence of SIRT3, the NMN rescue was completely abolished (Figure 4B–E). These results demonstrate that the NMN rescue in our model is exclusively dependent on the presence of SIRT3.

## 3. Discussion

The current study identifies an unrecognized role of KLF15 in regulating cardiac oxidative stress. We showed that KLF15 regulates cardiomyocyte ROS clearance by transcriptionally regulating NAMPT and cardiac NAD^+^, which then affects mitochondrial global acetylation and the activity of key antioxidants, including MnSOD. Combined with our previous discoveries on the role of KLF15 in cardiac lipid flux, we began to unveil the role of KLF15 in coupling lipid utilization and ROS clearance in the heart.

Coordinating the catabolism and its oxidative byproduct is likely of evolutionary advantage for the animal. However, in the case of chronic heart disease, KLF15 levels are reduced [5,11]. This not only affects energy production through catabolism, but also affects the ROS handling capacity, which may lead to increased vulnerability to ROS injuries. This has been demonstrated in cKlf15 KO, which has increased susceptibility to pressure overload and IR injury [9,10,11].

NAD supplementation has attracted attention in recent years, with over 900 clinical trials having been documented (clinicaltrials.gov assessed on 10 September 2021). However, the outcome has not been as exciting as what the preclinical studies promised. Several factors might be at play, e.g., the uncontrolled dosing and plasma or tissue levels, the effective dosing time window, and the appropriate target population and controls. Our study suggests that NAD^+^ supplementation may be particularly beneficial for patients with chronic heart disease, and who have low cardiac KLF15 levels. This information will be useful in future clinical trial designs and potentially help yield more promising therapeutic benefits in patients.

Previously, it has been demonstrated that the NMN protective effect in ischemia reperfusion is abolished in systemic Sirt1 KO mice [15]. Surprisingly, our results now suggest that SIRT3 is the main mediator, at least in the in vitro oxidative stress model. Further evaluation of the role of SIRT3 in mediating the NMN-mediated cardiac protection in vivo using cardiac-specific deletion models will be the next step in future studies.

In conclusion, we demonstrated that KLF15 regulates oxidative stress in the cardiomyocytes by regulating NAD^+^ levels through *Nampt*. Together with the growing recognition of its role in fatty acid utilization and fasting adaptation, we propose KLF15 as an evolutionarily conserved factor in the coordination of catabolism and ROS homeostasis in the heart.

## 4. Materials and Methods

### 4.1. Mice

All animal studies were approved by the Institutional Animal Care and Use Committee at Baylor College of Medicine (Institution number: AN-7226) and conducted in strict accordance with the NIH Guide for the Care and Use of Laboratory Animals. Mice were housed in a temperature- and humidity-controlled Specific Pathogen Free (SPF) facility with a 12 h light/dark cycle and ad libitum access to water and standard laboratory rodent chow. A total of 30 mice were used in the studies presented in this manuscript.

### 4.2. Cell Culture

Neonatal rat ventricular myocytes (NRVMs) were isolated from 2-day-old Sprague Dawley rats and cultured as described [24]. Isolated NRVMs were cultured for 48 h under quiescent conditions (DMEM supplemented with 0.1% bovine serum albumin, 1× insulin/transferrin/selenium, and 1% Penicillin/Streptomycin) prior to experiments. Following quiescence, NRVM were infected with adenoviral vectors (*sh-sc* or *sh-Klf15*) for 48 h prior to experiments. Adenoviral infection was carried out with the indicated virus (50 MOI for knockdown) for 48–72 h prior to experiments. For transient transfections, the cells were transfected with siRNA (25 nM) against rat Sirt1 (s234874, ThermoFisher, Waltham, MA, USA), Sirt3 (s147714, ThermoFisher, Waltham, MA, USA), or the negative control (4390843, ThermoFisher, Waltham, MA, USA), using the Viromer^®^ blue Transfection Reagent (VB-01LB-01, Lipocalyx, Halle, Sachsen-Anhalt, Germany).

### 4.3. Cytotoxicity Assay (LDH Assay)

After 48 h of infection with adenoviral vectors (*sh-sc* or *sh-Klf15*), NRVMs were challenged with different stressors (H_2_O_2_ for 2 h, ethanol for 6 h, 4HNE for 8 h). At the end of treatment, the supernatant was collected for LDH assay following the manufacturer’s instructions (Roche, Basel, Basel-Stadt, Switzerland). *n* = 4, data presented as mean ± SEM.

### 4.4. Oxygen Consumption Assay (Seahorse Assay)

Mitochondrial respiration rate in NRVMs was assessed using an Agilent Seahorse XFp Extracellular Flux Analyzer with the Mito Stress Test Kit (Agilent, Santa Clara, CA, USA). Pyruvate +glucose was used as a standard substrate to interrogate mitochondrial respiration capacity. NRVMs were seeded at a cellular density of 70,000 cells/well.

### 4.5. Immunoblot and Antibodies

Whole-cell lysates or isolated mitochondria lysates were prepared by homogenizing the basal regions of the hearts in the RIPA buffer (Invitrogen, Waltham, MA, USA) supplemented with protease inhibitors (Roche, Basel, Basel-Stadt, Switzerland). The blots were probed with the antibodies list below and normalized to VDAC (Cell Signaling Technology, Danvers, MA, USA) or GAPDH (Sigma, St. Louis, MA, USA). Anti-SOD2/MnSOD (acetyl K122) antibody (Abcam, Cambridge, Cambridgeshire, UK), anti-Superoxide Dismutase (MnSOD) (KC-19) antibody (Sigma, St. Louis, MA, USA), anti-SIRT3 antibody, anti-Acetylated-Lysine antibody (Cell Signaling Technology, Danvers, MA, USA), and anti-NAMPT antibody (ThermoFisher, Waltham, MA, USA).

### 4.6. NAD^+^ Measurement

Total NAD^+^ and nicotinamide adenine dinucleotide diaphorase (NADH) levels were measured using the NAD^+^/NADH Quantification Colorimetric Kit (BioVision Inc., Milpitas, CA, USA) following manufacturer’s instructions, and 2 × 10^5^ cells were used.

### 4.7. DHE Staining

NRVMs were treated with angiotensin II or H_2_O_2_ for the indicated time, then loaded with 5 μM DHE (Invitrogen, Waltham, MA, USA) following manufacturer’s instructions. Fresh mice heart tissue samples were snap frozen in OCT compound (ThermoFisher, Waltham, MA, USA). Cryopreserved sections were then loaded with 500 µM DHE following manufacturer’s instruction. Images were taken using fluorescent microscopy and analyzed with NIH Image J software.

### 4.8. Quantitative RT-PCR

RNA from NRVM were isolated using Quick-RNA (Zymo Research, Irvine, CA, USA) according to manufacturer’s directions. RNA was reverse transcribed using iScriptTM (Biorad, Hercules, CA, USA) following manufacturer’s protocol. Quantitative PCR was performed with the TaqMan method on a QuantStudio™ 5 Real-Time PCR System (Applied Biosystems, Waltham, MA, USA). Relative expression was calculated using the ^ΔΔ^Ct method, with normalization to Gapdh. Specific primer/probe sequences are available upon request.

### 4.9. Statistics

Results are presented as mean ± SEM. Two-tailed Student’s *t*-test was used to compare the difference between two groups. Two-way ANOVA with Bonferroni correction was used for multiple comparisons. Statistical significance was defined as *p* < 0.05.

## Data Availability

The data presented in this study are available in “KLF15 Regulates Oxidative Stress Response in Cardiomyocytes through NAD^+^”.
